# Importance of Android/Gynoid Fat Ratio in Predicting Metabolic and Cardiovascular Disease Risk in Normal Weight as well as Overweight and Obese Children

**DOI:** 10.1155/2014/846578

**Published:** 2014-09-15

**Authors:** Lennie Samsell, Michael Regier, Cheryl Walton, Lesley Cottrell

**Affiliations:** ^1^Department of Pediatrics, School of Medicine, West Virginia University, Morgantown, WV 9214, USA; ^2^Department of Biostatistics, School of Public Health, West Virginia University, Morgantown, WV 6057, USA

## Abstract

Numerous studies have shown that android or truncal obesity is associated with a risk for metabolic and cardiovascular disease, yet there is evidence that gynoid fat distribution may be protective. However, these studies have focused on adults and obese children. The purpose of our study was to determine if the android/gynoid fat ratio is positively correlated with insulin resistance, HOMA2-IR, and dislipidemia in a child sample of varying body sizes. In 7–13-year-old children with BMI percentiles ranging from 0.1 to 99.6, the android/gynoid ratio was closely associated with insulin resistance and combined LDL + VLDL-cholesterol. When separated by sex, it became clear that these relationships were stronger in boys than in girls. Subjects were stratified into BMI percentile based tertiles. For boys, the android/gynoid ratio was significantly related to insulin resistance regardless of BMI tertile with and LDL + VLDL in tertiles 1 and 3. For girls, only LDL + VLDL showed any significance with android/gynoid ratio and only in tertile 2. We conclude that the android/gynoid fat ratio is closely associated with insulin resistance and LDL + VLDL-, “bad,” cholesterol in normal weight boys and may provide a measurement of metabolic and cardiovascular disease risk in that population.

## 1. Introduction

Childhood obesity is a common health problem in the United States and despite public focus addressing the problem, obesity rates among school-age children (6–19 years old) remain high at 19%. An additional 25% of children are overweight, increasing the concern and need to continue obesity prevention and treatment efforts nationwide [1]. Obesity in children is not only a risk factor for adult cardiovascular and metabolic disease but may also predict pediatric onset of heart disease and type 2 diabetes [2, 3]. Researchers have also established that earlier and longer durations of obesity throughout childhood increase one's risks of these chronic conditions in adulthood [4, 5].

Childhood and adult obesity can come in many different forms that are not inherently equal in terms of their health impact. The existing literature reflects that truncal adiposity, or the android body type, is a strong indicator of risk for disease [6–8]. Although the relative importance of subcutaneous versus visceral fat for risk is controversial [9–11], it is generally accepted that android obesity is an important risk factor for insulin resistance. Lower extremity adiposity, or the gynoid body type, may even lower that risk [12].

Insulin plays a crucial role in metabolism, and insulin resistance may be the underlying linkage between obesity, type 2 diabetes, and cardiovascular disease [13]. Much of the literature addressing the association between fat deposition and insulin resistance has been focused on android obesity alone, often using waist circumference or skinfold measurements to represent abdominal obesity. However, recent applications of dual X-ray absorptiometry (DXA) [14–17] have also allowed us to assess various regions of fat deposition and determine the android/gynoid fat ratio.

Studies have shown important relationships between the android/gynoid ratio and metabolic [18, 19] and cardiovascular disease [20] risks in healthy adults. A few studies have also shown this association in children [21, 22] but only in overweight or obese children. Still very little is known about the relationship between the android/gynoid ratio to cardiovascular or metabolic risk in normal weight children. Our objective was to determine the association of android/gynoid fat ratio with insulin resistance and dislipidemia, irrespective of child obesity. This work is significant because it may provide an assessment of metabolic and cardiovascular disease risk in children without the obvious risk of obesity and provide healthcare providers with an early tool to aid in the prevention of these disease states.

## 2. Methods

### 2.1. Ethical Considerations

All study protocols were reviewed and approved by the West Virginia University Institutional Review Board prior to any research activity. Signed parental consents and child assents were obtained from all participants before collecting any surveys or performing any examination of the subjects.

### 2.2. Study Participants

Children aged 7 to 13 years were either recruited from outpatient pediatric clinics or as a follow-up to their participation in an annual school screening program in a rural, Eastern United States setting. Study flyers were posted within the clinics and throughout the communities (e.g., grocery stores and schools) and were sent home with the screening program participants. The cross-sectional study design using the aforementioned recruitment strategy over the study period, 24 months, enrolled 73 children participants.

### 2.3. Procedures

Families who contacted the research team with an interest to participate in the study scheduled a clinic visit in the Physician Office Center—Pediatric and Adolescent Group Practice (PAGP) in Morgantown, West Virginia. Basic contact information was collected so that a series of surveys for one parent or legal guardian and the child could be mailed prior to the scheduled visit. Families were instructed to return completed surveys on the day of their appointment. Children were also asked to fast overnight before the visit and to abstain from any medications as appropriate.

Upon arriving at the clinic visit, anthropometrics were obtained and recorded for the child without shoes. Because the child was fasting, up to 15 cc of serum was drawn from each participant. Once the child was able to eat a breakfast, which was provided, a urine sample was collected and a pulmonary function test and allergy testing were completed. Parents were asked to complete additional surveys related to their child's health and medical history while the clinical assessments were being conducted. The clinic visit took an average of three hours to complete. Families were reimbursed for their travel and time.

### 2.4. Measures

*Child Body Mass Index (BMI) Percentile.* Children's height and weight measurements were used to calculate a raw BMI and BMI percentile score using the Centers for Disease Control and Prevention formula: weight (lb)/(height (in))^2^ × 703 [23]. We then calculated the BMI-for-age percentile using recommended age- and sex-specific growth charts [24].

Total body, android, and gynoid fats were measured using an iDXA X-ray bone densitometer (GE Healthcare) with software designed for a pediatric population. Android was measured with the lower boundary at the pelvis cut with the upper boundary above the pelvis cut by 20% of the distance between the pelvis and neck cuts. Gynoid upper boundary was below the pelvis cut line by 1.5 times the android space and gynoid space was equal to 2 times the android space [25]. Total body fat was expressed as percent of total body mass and android and gynoid were expressed as percent of total body fat. Android/gynoid ratio was android fat divided by gynoid fat.

### 2.5. Biochemical Analyses

Blood samples were collected in a Red/Black SST tube and a K2 EDTA tube using standard venipuncture practices. The SST tube sat at room temperature for 30 minutes followed by centrifugation at 1000 rcf for 10 minutes at room temperature. The samples were taken to LabCorp. for testing of insulin, glucose, total cholesterol, HDL-cholesterol, LDL-cholesterol, VLDL-cholesterol, and triglycerides.

HOMA2-IR was used to assess insulin resistance from fasting glucose and insulin levels, (http://www.dtu.ox.ac.uk/homacalculator/). This computer generated homeostasis model assessment has been shown to correlate closely with the euglycemic clamp model [26].

### 2.6. Statistics

Means, standard errors, and *t*-tests, by child's sex, were generated for subject characteristics. Simple and multivariable linear regression was used to assess the association between BMI Z score, percent of total body fat or android/gynoid ratio, age, and sex with the disease risk factors: HOMA2-IR, plasma total cholesterol, HDL-cholesterol, combined LDL + VLDL-cholesterol, and triglycerides. BMI tertiles, based on the BMI percentiles, were used to further explore the aforementioned associations.

We assessed the associations using the adjusted coefficient of multiple determination which adjusts for the number of variables in the model and ameliorates problems with artificial inflation of the *R* ^2^ when more than one variable is included in the model. To measure the conditional reduction in the variation of the responses (disease risk factors) when additional variables are added to the model, we used the coefficient of partial determination. This measures the additional proportion of explained variation when a new variable is used in the model given that other variables are already in the model [27]. All statistical tests assumed a 0.05 alpha-level; JMP (SAS, Inc., Cary, NC) and *R* [28] statistical software were used for the analyses.

## 3. Results

Table 1 shows the age and anthropometric and biochemical characteristics of our study population, separated by sex and combined. Characteristics of boys and girls were very similar in our population. Percent of gynoid fat was the only measurement of interest having a sex difference, with girls having a slightly higher value than boys, *P* < 0.05. Percent of android fat also tended to be higher in girls leading to a very similar android/gynoid ratio between the sexes.

We used simple linear regression to assess the relationship between three different covariates of obesity (BMI Z score, percentage of total body fat and android/gynoid ratio) and the outcomes of cardiovascular and metabolic risk factors (Table 2). We observed a general pattern of statistical significance for a linear relationship between each outcome-covariate pair across the models. We observed that the android/gynoid ratio had the strongest relationship with all factors. This was particularly true for HOMA2-IR, with the android/gynoid ratio explaining almost 46% of the variation for our measure of insulin resistance. Of all lipids, combined LDL + VLDL had the strongest relationship with all of our measures of obesity and again the strongest was with android/gynoid ratio.

Our emphasis on the android/gynoid ratio (Table 3) revealed that HOMA2-IR was significantly related to the android/gynoid ratio in both girls (*R* ^2^-adj = 0.322, *P* value < 0.001) and boys (*R* ^2^-adj = 0.523, *P* value < 0.001). Considering the adjusted coefficient of multiple correlations, we observed adjusted *R* ^2^ values that were between those of the girls and the boys. HOMA2-IR was particularly strong for both the sex adjusted model (*R* ^2^-adj = 0.452, *P* value < 0.001) and the sex and age adjusted model (*R* ^2^-adj = 0.508, *P* value < 0.001). Again, LDL + VLDL was the lipid most affected by the android/gynoid ratio, particularly in boys.

The coefficient of partial determination measures the marginal contribution of one covariate when others are already in the model. We observed that when the android/gynoid ratio is already in the model, the addition of the child's sex only reduced residual sum of squares by 0.02%. In contrast, when the android/gynoid ratio is already in the model, the addition of the child's age provided an 11.54% reduction in the residual sum of squares. We noted that if both the android/gynoid ratio and age are already in the model, the addition of sex decreased the residual sum of squares by only 0.09%. We noted that, in all our sex adjusted regression models, the model coefficient for the sex covariate was not statistically significant, despite the differences in significance of *R* ^2^ between sexes in all lipid measures.

Finally, we assessed if the relationships between the outcomes and the android/gynoid ratio were being driven by the most obese of our subjects, by considering the android/gynoid ratio relationships within tertiles based on BMI percentile. We observed that there is a 5.9% reduction in the residual sum of squares with the addition of BMI tertiles to the regression model when the android/gynoid ratio is already in the model. Furthermore, we had an 11.9% reduction in the residual sum of squares with the addition of age when the BMI tertiles are already in the model. Finally, we observed that there was a 6.3% reduction in the residual sum of squares with the addition of BMI tertiles to the regression model when the android/gynoid ratio and age were already in the model. Based on these results, we explored both within BMI tertile strata correlations and adjusted correlations (Table 4).

The differences between sexes became more apparent in these analyses. For girls, we observed a statistically significant association between VLDL + LDL-cholesterol and the android/gynoid ratio within BMI tertile 2 (*R* ^2^-adj = 0.335, *P* value = 0.038) and a statistically significant association between HOMA2-IR, adjusted for BMI and age, and the android/gynoid ratio (*R* ^2^-adj = 0.555, *P* value < 0.001). There are no other significant associations in girls.

For boys, we observed that, across all three tertiles, the android/gynoid ratio was significantly associated with HOMA2-IR (Table 4). An android/gynoid ratio and triglycerides association was observed for the BMI tertile 1 (*R* ^2^-adj = 0.225, *P* value = 0.049) and with the model adjusted for BMI and age (*R* ^2^-adj = 0.197, *P* value = 0.003). A statistically significant relationship between the ratio and total cholesterol was observed for BMI tertile 3 (*R* ^2^-adj = 0.362, *P* value = 0.014) and with the model adjusted for BMI and age (*R* ^2^-adj = 0.254, *P* value = 0.001). Tertiles 1 and 3 had significant relationships between the android/gynoid ratio and VLDL + LDL-cholesterol (Table 4). Finally, the models for HDL- and VLDL + LDL-cholesterol, when adjusted for BMI and age, had significant associations with the android/gynoid ratio (Table 4).

## 4. Discussion

In our population of 7–13-year-old boys and girls, the android/gynoid ratio proved to be the obesity measure most closely related to both insulin resistance and dislipidemia. An important and unique observation was that the relationship between both metabolic and cardiovascular disease risk and android/gynoid ratio was strong in normal weight boys as well as the overweight or obese. These relationships did not hold true for girls.

### 4.1. Body Shape and Sex in Disease Risk

When compared to BMI Z score and percent of total body fat, we found that the android/gynoid ratio was clearly the most closely related to all disease risk factors. However, the effects of android/gynoid ratio on HOMA2-IR did differ by age. In boys, all risk factors showed a significant relationship with the android/gynoid ratio, and HOMA2-IR and LDL + VLDL-cholesterol had very high correlations, while, in girls, only HOMA2-IR was significantly related to the android/gynoid ratio. Also, of note, the effect of age as a covariate was lost in males but not females.

In 1996, Vague [29] noted a sex difference in fat deposition and a worse metabolic profile in the male (android) than the female (gynoid) body type. Numerous studies have tested and substantiated these findings in adults. However, sex differences in fat deposition and other cardiovascular and metabolic disease risk factors are not as evident in children [30, 31]. Studies have shown differences, but the findings are not consistent and often not apparent until puberty [32, 33]. The varying effects of sex and age in our study may be confounded by the age of our population, where girls are closer to puberty than boys and hormonal changes may already be occurring [32].

### 4.2. Importance of Android/Gynoid Ratio Even without Obesity

In our subjects, the android/gynoid ratio was a good predictor of both insulin resistance and the cardiovascular risk factor, LDL + VLDL-cholesterol, in normal as well as overweight or obese boys. The BMI percentile of our population ranged from 0.1 to 99.6 percentile, providing us with an opportunity to assess the relationship between android/gynoid ratio and disease risk in normal weight children as well as overweight and obese. When we broke our subjects into tertiles of BMI percentile, the android/gynoid ratio in boys was significantly correlated with HOMA2-IR regardless of BMI tertile and with LDL + VLDL-cholesterol in both the low and high tertiles. However, the effect of android/gynoid ratio on HOMA2-IR in girls was lost.

Various anthropometric measurements have been used to assess metabolic and cardiovascular risk, including BMI and percent body fat, as well as site specific measurements, such as abdominal or android fat and waist circumference. A number of studies have shown that high levels of central or truncal obesity carry risks for both metabolic and cardiovascular diseases in adults [6, 11, 34] and children [35–37]. Similar to our study, Aucouturier et al. [21] showed that the android/gynoid ratio was a significant predictor of HOMA2-IR in children and adolescents. However, that study was only in overweight and obese subjects, while our population included normal weight children.

### 4.3. Opportunities and Implications

The fact that the android/gynoid ratio is related to disease risk, even if a child is at normal weight, gives us a target area for changes in body fat. Interventions in both children and adults have been shown to decrease android fat and improve insulin resistance. Tang et al. [38] found that a moderately low calorie but high protein diet lowered android fat mass in adults and Keating et al. [39] showed that continuous exercise but not high intensity interval training lowered android fat in adults. Aerobic exercise and resistance or strength training have been found to be effective in reducing both android fat [40, 41] and insulin resistance [42, 43] in children and adolescents. Future pediatric studies should include serial measurements of android/gynoid ratio or the substitute, waist/hip ratio, as well as other metabolic and cardiovascular disease risk factors, combined with either diet or exercise interventions.

### 4.4. Limitations

Our study was cross-sectional rather than longitudinal, limiting what can be said about cause and effect between the android/gynoid ratio and disease risk. Also, we made no measures of Tanner stages and cannot assess the impact that puberty may be having on our findings, particularly those of sex and age. The sample size restricted our ability to explore more complex relationships such as covariate interactions.

### 4.5. Conclusion

The finding that the android/gynoid ratio is highly correlated to risk factors for both metabolic and cardiovascular diseases in normal weight boys is important. It is impractical to expect that DXA scans will become a part of routine screenings in apparently healthy children. However, Arnberg et al. [22] found that the waist/hip ratio was highly correlated with the android/gynoid ratio in children and may offer a convenient alternative. This could provide healthcare providers with a simple noninvasive tool to assess which children are at risk and should be included in more comprehensive testing for metabolic and cardiovascular diseases.

## Figures and Tables

**Table 1:** Characteristics, anthropometrics, and biochemistries of participants, stratified by sex and combined (mean ± SE).

Variable	Girls (*n* = 33)	Boys (*n* = 40)	Total (*n* = 73)
Anthropometrics and age			
Age (years)	9.5 ± 0.3	9.6 ± 0.3	9.5 ± 0.2
BMI *Z* score	0.43 ± 0.19	0.47 ± 0.21	0.46 ± 0.14
BMI percentile	63 ± 5	61 ± 5	62 ± 3

BMI tertiles (percentiles)			
1 mean ± SE	28 ± 4	24 ± 4	26 ± 3
(min–max)	(0–44)	(0–43)	(0–44)
2 mean ± SE	66 ± 3	66 ± 4	66 ± 2
(min–max)	(45–82)	(47–83)	(45–83)
3 mean ± SE	93 ± 1	95 ± 2	94 ± 1
(min–max)	(85–98)	(85–100)	(85–100)

DXA measurements			
Total body fat (%)	31.9 ± 1.4	29.6 ± 1.6	30.5 ± 1.1
Android body fat (% of total)	27.5 ± 2.2	24.9 ± 2.2	25.9 ± 1.7
Gynoid body fat (% of total)	37.0 ± 1.4	32.7 ± 1.4	34.6 ± 1.0
Android/gynoid ratio	0.71 ± 0.04	0.70 ± 0.04	0.70 ± 0.03

Blood constituents			
Glucose (mmol/L)	4.66 ± 0.07	4.80 ± 0.05	4.73 ± 0.04
Insulin (pmol/L)	53.2 ± 4.6	51.1 ± 5.2	52.0 ± 3.52
HOMA2-IR	1.13 ± 0.10	1.09 ± 0.11	1.11 ± 0.07
Triglycerides (mmol/L)	1.17 ± 0.23	0.90 ± 0.10	1.00 ± 0.12
Total cholesterol (mmol/L)	4.04 ± 0.09	4.27 ± 0.11	4.17 ± 0.07
HDL-cholesterol (mmol/L)	1.38 ± 0.06	1.51 ± 0.06	1.45 ± 0.04
LDL + VLDL-cholesterol (mmol/L)	2.56 ± 0.10	2.76 ± 0.12	2.67 ± 0.08

**Table 2:** Regression based correlation analyses adjusted *R* ^2^ for each outcome, HOMA2-IR, triglycerides, total cholesterol, and HDL-cholesterol and combined LDL + VLDL-cholesterol and each covariate BMI *Z* score, total body fat, and android/gynoid ratio.

Outcome	Covariate		
	BMI *Z* score	Percent of total body fat	Android/gynoid
	*R* ^2^-adj (*P* value)	*R* ^2^-adj (*P* value)	*R* ^2^-adj (*P* value)
HOMA2-IR	0.276 (<0.001)	0.293 (<0.001)	0.459 (<0.001)
Triglycerides	0.039 (0.050)	0.039 (0.052)	0.063 (0.018)
Total cholesterol	0.055 (0.026)	0.085 (0.007)	0.088 (0.006)
HDL-cholesterol	0.079 (0.009)	0.044 (0.041)	0.103 (0.003)
LDL + VLDL-cholesterol	0.185 (<0.0001)	0.199 (<0.001)	0.268 (<0.001)

**Table 3:** Regression based correlation analyses adjusted *R* ^2^ between android/gynoid ratio and our metabolic and cardiovascular risk factors. Regression models for each sex as well as a sex adjusted and a sex and age adjusted regression model.

Outcome	Android/gynoid			
	*R* ^2^-adj (*P* value)			
	Girls	Boys	Sex adjusted model	Sex and age adjusted model
HOMA2-IR	0.322 (<0.001)	0.523 (<0.001)	0.452 (<0.001)	0.508 (<0.001)
Triglycerides	0.009 (0.267)	0.195 (0.003)	0.060 (0.018)	0.068 (0.018)
Total cholesterol	<0.001 (0.817)	0.223 (0.001)	0.113 (0.006)	0.121 (0.005)
HDL-cholesterol	0.002 (0.311)	0.156 (0.007)	0.114 (0.003)	0.124 (0.003)
LDL + VLDL-cholesterol	<0.001 (0.761)	0.438 (<0.001)	0.270 (<0.001)	0.261 (<0.001)

**Table 4:** Regression based correlation analyses adjusted *R* ^2^ between android/gynoid ratio and our metabolic and cardiovascular risk factors. Regression models for each BMI tertile as well as a BMI tertile and age adjusted regression model.

	Android/gynoid ratio *R* ^2^-adj. (*P* value)			
	BMI 1st tertile	BMI 2nd tertile	BMI 3rd tertile	BMI and age adjusted
Both sexes				
HOMA2-IR	0.307 (0.003)	0.111 (0.057)	0.386 (0.001)	0.532 (<0.001)
Triglycerides	0.053 (0.144)	<0.001 (0.439)	<0.001 (0.464)	0.060 (0.018)
Total cholesterol	0.095 (0.078)	<0.001 (0.544)	0.100 (0.073)	0.107 (0.006)
HDL-cholesterol	<0.001 (0.564)	<0.001 (0.545)	0.079 (0.099)	0.087 (0.004)
LDL + VLDL-cholesterol	0.146 (0.037)	0.034 (0.187)	0.266 (0.007)	0.268 (<0.001)

Female^a^				
HOMA2-IR	0.330 (0.082)	0.065 (0.400)	0.261 (0.131)	0.555 (<0.001)
Triglycerides	0.010 (0.785)	0.060 (0.421)	0.001 (0.924)	0.086 (0.280)
Total cholesterol	0.047 (0.549)	0.097 (0.301)	0.303 (0.099)	0.028 (0.824)
HDL-cholesterol	0.106 (0.359)	0.034 (0.544)	0.032 (0.622)	0.053 (0.331)
LDL + VLDL-cholesterol	0.006 (0.836)	0.335 (0.038)	0.268 (0.153)	0.039 (0.769)

Male^b^				
HOMA2-IR	0.256 (0.037)	0.449 (0.017)	0.402 (0.009)	0.538 (<0.001)
Triglycerides	0.225 (0.049)	0.002 (0.894)	0.200 (0.062)	0.197 (0.003)
Total cholesterol	0.174 (0.077)	0.004 (0.842)	0.362 (0.014)	0.254 (0.001)
HDL-cholesterol	0.028 (0.264)	0.000 (0.958)	0.118 (0.123)	0.246 (0.005)
LDL + VLDL-cholesterol	0.281 (0.030)	0.011 (0.751)	0.563 (0.002)	0.408 (<0.001)

^a^ *R* ^2^ is reported because the *R* ^2^-adj produced negative values. Negative values can occur when using the *R* ^2^-adjusted if the model contains terms that do not help to predict the response [44].

^b^ *R* ^2^ is reported for BMI 2nd tertile because the *R* ^2^-adj produced negative values.
